# Genetic Evidence of Multiple Introductions of Lumpy Skin Disease Virus into Saratov Region, Russia

**DOI:** 10.3390/pathogens10060716

**Published:** 2021-06-07

**Authors:** Yuri V. Saltykov, Anna A. Kolosova, Nadezhda N. Filonova, Alexander N. Chichkin, Valentina A. Feodorova

**Affiliations:** Laboratory for Molecular Biology and NanoBiotechnology, Federal Research Center for Virology and Microbiology, Branch in Saratov, 410028 Saratov, Russia; yury.saltykov@mail.ru (Y.V.S.); kolosova_anna_a@mail.ru (A.A.K.); nadezhda-filonova@bk.ru (N.N.F.); alexander.chichkin@inbox.ru (A.N.C.)

**Keywords:** capripoxvirus, lumpy skin disease, GPCR, livestock, typing, LSDV lineage, heterologous vaccine

## Abstract

Lumpy skin disease virus (LSDV) is the causative agent of lumpy skin disease (LSD) that has been recently reported in the South-East and North Asian parts of the Russian Federation. During 2017–2019, there were more than 30 LSD outbreaks in Saratov Region despite active inoculation of cattle with heterologous vaccine. Importantly, the first case of the novel recombinant LSDV strain was reported here in 2017. This study aimed to determine the main clonal lineage(s) of LSDV strains circulated within Saratov Region and other regions of Russia since the first introduction of LSDV. The molecular typing and subtyping based on the coding regions of the G-protein-coupled chemokine receptor (GPCR) gene resulted in a discrimination of all outbreak-related LSDV strains into two main types, such as Type I and Type II, and subtypes Ia-d and IIa-g. Phylogenetically, eleven LSDV lineages were revealed in Russia including the five ones in Saratov Region. They were the following: (i) the Neethling wild Type Ia/2017; (ii) the recombinant Saratov IIc/2017/2019; (iii) the specific Dergachevskyi IId/2017; (iv) the Khvalynsky IIg/2018, and (v) the Haden-Type IIa lineage for the six LSDV strains detected in cattle immunized with heterologous vaccine during the last LSD outbreak in the Saratov Region, Nesterovo Village, in 2019 (Nesterovo-2019 strains). A single LSDV strain detected in Saratov Region in 2017 had the same Type Ia that was identified in 2016 in the bordered Republic of Kazakhstan. Phylogeographic analysis demonstrated three nominal clusters of LSDV types in the following Russian Federation territories: (I) the Central European part; (II) the South-East of the European part; (III) the North Asian part. Cluster I was represented by mainly Type I strains, while both Clusters 2 and 3 contained predominantly Type II strains. The Clusters I and II partially overlapped, while Cluster 3 was separate. Multiple introductions of LSDV into Saratov Region in 2017–2019 using GPCR-based molecular typing and subtyping were revealed. This scheme is a promising tool for molecular discrimination of LSDV strains derived from both vaccinated and unvaccinated against LSD cattle as well as for molecular epidemiology.

## 1. Introduction

Lumpy skin disease virus (LSDV), the causative agent of lumpy skin disease (LSD) is known as one of the three species, namely LSDV, sheeppox virus (SPPV) and goatpox virus (GTPV) in the genus Capripoxvirus (CapPVs) within the Poxviridae family [1,2,3,4,5,6,7,8,9,10]. The LSDV is a relatively large, double-stranded DNA enveloped virus with a genome of 151-kbp that contains 156 putative genes sharing 97% nucleotide identity with the genomes of two other CapPVs, SPPV and GTPV [7,11]. All SPPV and GTPV genes have been found in LSDV [12] providing the marked serologic cross-reactivity between these three CapPVs [2]. LSD is an emerging transboundary disease with a marked morbidity rate of 3–85% [6,8,13] and mortality of 40–75% in naïve cattle populations [6,14]. LSD outbreaks produce wide economic losses due to decreased milk and beef yield, abortions and reduced quality of bovine semen material [7,9,15,16] resulting in a great impact on both national and world livestock industry. Moreover, quarantine measures and outbreak management carry additional costs.

The first case of the disease was registered more than 90 years ago, in 1929, in sub-Saharan Africa [17]. From there, the infection dramatically spread, first, to most parts of Africa, and then through the Middle East and further to a number of countries in the European and Asian part of the Eurasian continent [1,6]. Since 2016, LSD outbreaks have been registered in many countries of Southwest Asia and Eastern Europe such as Turkey, Greece, Bulgaria, the Republic of North Macedonia, Serbia, Kosovo, Albania, Montenegro, the Russian Federation, Armenia, Georgia and Kazakhstan, followed by a spread to a few new countries of East Asia, namely China, Bangladesh and India [1,10,18,19]. Despite the fact that LSD is considered to be a global emerging worldwide threat in Europe, the Middle East and Asia [5,6,10], it is a vaccine-controlled disease.

Live attenuated homologous and heterologous vaccines against LSD based on attenuated strains of CapPVs, which originated from wild field isolates, have been successfully developed. However, their efficacy in the field has not been fully evaluated. Homologous vaccines are mostly based on the LSDV Neethling strain isolated in South Africa in 1950s. Their efficacy was proven by the absence of any LSD outbreaks in Southeastern Europe after the annual mass vaccination of cattle in all affected countries during the last three years [1]. Heterologous vaccines were generally developed with the use of either SPPV or GTPV strains such as Yugoslavian RM-65 and Romanian SPPV strains etc. [1,20]. Theoretically, this type of vaccine could provide protective immunity against all CapPVs-related infections, including LSD. Such cross-protectivity might be possible since the strains of CapPVs were proven to be antigenically indistinguishable. Moreover, it was reported [21] that recovery from infection with one strain provided immunity against all other strains. Thus, taking into account a marked antigenic homology across the strains of CapPVs, a single strain based vaccine could have a good protective potency in eliciting protective immunity in both cattle and small ruminants [21]. However, in cattle both types of these vaccines were in fact associated with incomplete protection against LSD [22,23] and post-vaccination adverse reactions resembling disease symptoms [22,24]. Moreover, LSD outbreaks have been registered in animals previously immunized with homologous live attenuated vaccines, which raised serious concerns with regard to differentiation of infected and vaccinated animals [24,25].

To discriminate wild field type LSDV strains derived from clinical cattle samples and LSDV vaccine strains, several different methods have been developed and successfully applied [24,26,27,28]. For this, LSDV target genome regions for the molecular detection and differentiation of these two types of LSDV strains were carefully selected. The genes were the following: the RPO30 gene encoded the RNA polymerase 30 kDa subunit, which plays a role in viral replication [29], the LSDV126 putative extracellular enveloped virus (EEV) and LSDV127 hypothetical glycoprotein genes [27,30]. The G-protein-coupled chemokine receptor (GPCR) gene that is involved into host immunomodulation [4] demonstrated greater nucleotide polymorphism in comparison with the sequences of these three critical genes, the RPO30, LSDV126 and LSDV127 [24,31].

A comparative analysis of the GPCR gene sequences of LSDV from wild field isolates and vaccine strains revealed the presence of a 12-bp deletion specific to the wild type of LSDV genomes that was absent in vaccine strains [4,32,33]. This finding highlighted the GPCR gene as the important candidate target for the development of a ‘DIVA’ (differentiation of infected from vaccinated animals) method for the precise detection of LSDV infections within herds immunized with homologous vaccines [24,25,26]. More recently, it was shown that the GPCR could be a suitable target for the genetic discrimination between members of Capripoxvirus genus [4,11,12]. In fact, a phylogenetic analysis based on the nucleic acid sequences of CapPVs isolates using the Neighbor-Joining algorithm showed three separate genetic clusters consisting of LSDV, GTPV and SPPV lineages [4]. Moreover, a certain intra-group diversity in the LSDV cluster was revealed [4,22,24,31,33,34,35,36,37,38,39,40]. Furthermore, a phylogenetic analysis clustered LSDV wild field outbreak-related isolates, vaccine and the so-called ‘vaccine-like’ strains into separate clades within the Capripoxvirus family [31,35,36,37,38,39,40].

The question is whether the GPCR sequence comparison is able to discriminate the clonal lineages of LSDV circulating through different world regions following the intra-specific typing and sub-typing of LSDV outbreak-associated isolates. Obviously, it could be an excellent tool for understanding LSDV molecular epidemiology, one that would help to improve our knowledge of enhanced control strategies at regional, national and world-wide levels. This tool could be used to understand the specific diversity of LSDV strains during both single and recurrent outbreaks and retrospectively, to identify the origin of the main lineage(s) of the pathogen related to the outbreak as the possible causative agent of LSD, and to unravel the case(s) of probable transboundary LSDV circulation and transmission to neighboring region(s). Further, it is important to unravel the molecular diversity of the so-called ‘vaccine-like’ LSDV strains, which were recently identified in some transboundary territories after the immunization of cattle against LSD with live homologous virus vaccines [31,36,37,39,40,41].

Since 2015, the World Organization for Animal Health (OIE) has reported more than 400 outbreaks of LSD in the Russian Federation [42]. There were two independent epidemic waves in which at least two main types of the LSDV isolates were recently identified: (i) field isolates only (2015–2016), and (ii) the so-called ‘vaccine-like’ variants (2017–2019), which genetically differed from the isolates of the first wave, as well as from other known field strains [31]. It was proposed that the latter variants represent a new emergence, rather than the continuation of the initial epidemic caused by the field-type strains [31]. However, it is not clear whether these ‘novel’ ‘vaccine-like’ variants are identical to each other and represent a single phylogenetic group of LSDV strains, or whether they consist of heterologous variants of different subtypes formally combined within the same phylogenetic cluster. Moreover, the next question is whether these ‘novel’ strains have any relation to previously identified isolates of LSDV, or whether they represent separate lineages that were not detected prior to 2017. The final question is whether it is possible to find these strains in samples taken exclusively from the vaccinated cattle, or they could be detected in unvaccinated animals too. In fact, both field LSDV and vaccine strains were isolated from cattle after their emergency vaccination against LSD with live homologous vaccines [24,25,43]. However, it is still unknown whether outbreak-associated or vaccine-related strains could be detected in cattle immunized with heterologous vaccines. Overall, monitoring the LSDV strains diversity is crucial for understanding both the virus evolution and the origin of outbreak emergence, as well as for evaluating vaccine quality and to assessing the risk for animal health after the introduction of different type of vaccines against LSD.

Since 2017 in the Russian Federation herds have been actively immunized with live heterologous vaccines against LSD based on sheep pox vaccine strain (SPPV vaccine) in the affected regions with 80–100% coverage in the vaccinated area [1]. There is total annual vaccination of all cattle in the Russian regions with a marked level of LSD morbidity, especially if the territory borders with countries in which LSD cases were reported or LSDV was detected either in the past or at present. One of such regions is Saratov Region, which borders the Republic of Kazakhstan where the LSDV field strains were reported in 2016 [44]. In fact, in Saratov Region at least 30 LSD outbreaks were officially registered during 2017–2019 [45]. Importantly, the first recombinant “novel” LSDV strains were isolated here in 2017 following repeated cases of their detection in both Saratov and other regions of the Russian Federation [31,36,40]. However, more information is needed on the biodiversity of the strains causing LSD outbreaks in this region, as well as for unraveling their possible relationship with other well-known LSDV lineage(s) of outbreak-associated strains.

The aim of this study was: (i) to conduct molecular typing of the LSDV strains detected in cattle immunized with SPPV vaccine during the last LSD outbreak in Saratov Region in September–October, 2019; and (ii) to determine the main clonal lineage(s) of LSDV strains circulating within Saratov Region in comparison with those detected in the Russian Federation since the first introduction of LSDV.

## 2. Results

### 2.1. Detection of LSDV DNA in Samples from Cattle Vaccinated and Unvaccinated with SPPV-Based Vaccine

Preliminarily, LSDV DNA was easily detected by means of commercial PCR in each of the two clinical samples, namely the blood and nasal discharge specimens derived from both naturally infected Case1 and Case2 vaccinated with SPPV-based vaccine. No SPPV/GTPV DNA was revealed in Case1 and Case2 when GPCR gene targeting PCR was applied. Similarly, Case3 demonstrated a positive response to LSDV DNA and negative PCR reaction for the presence of SPPV/GTPV DNA in either blood or nasal specimens. No CapPVs genetic material was found in the specimens from Cow4 (Table 1).

### 2.2. Seroconversion in Cattle Vaccinated with SPPV-Based Vaccine

All cattle vaccinated with SPPV-based vaccine, namely Case1, Case2 and Cow4 were positive in ELISA for the presence of LSDV specific antibodies/CapPVs specific antibodies. The unvaccinated cow of Case3 showed a negative response in this test (Table 1).

### 2.3. Phylogenetic Analysis and Typing and Subtyping

**Figure 1:**
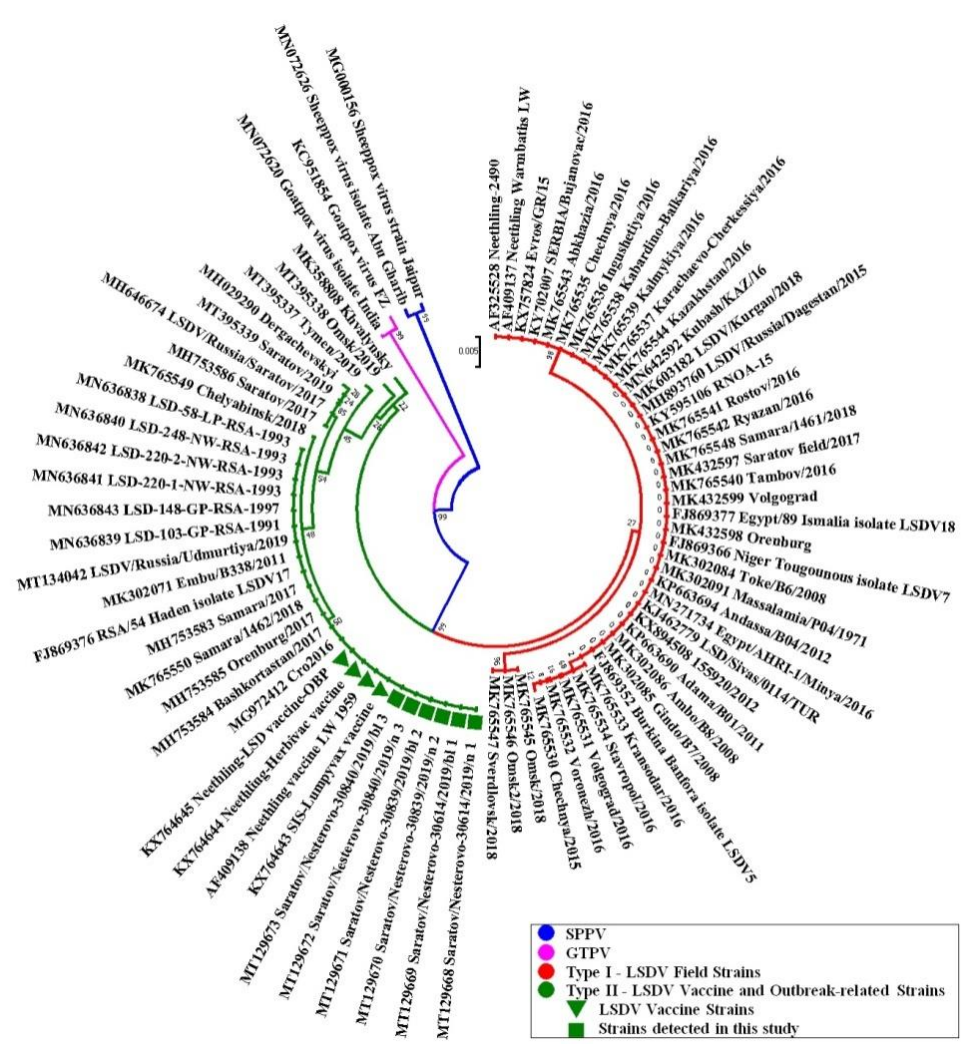
Phylogenetic tree demonstrating the relationship between the LSDV GPCR gene sequences derived from the LSDV strains detected in Saratov Region in 2019 (Nesterovo-2019) and the GPCR gene sequences of other CapPVs available in NCBI database and listed in Table 2 and Table S1 in Supplementary Material. The tree was generated using the Neighbor-Joining method in MEGA 7 [46] with branch lengths in the same units as those of the evolutionary distances used to infer the phylogenetic tree. The evolutionary distances were computed using the Maximum Composite Likelihood method and are in the units of the number of base substitutions per site. The sequences for different CapPVs spp. are highlighted as the color circles: blue for SPPV, pink—for GTPV, red and& green—for LSDV of Type I and Type II, respectively. The LSDV vaccine strains of the Neethling-Type are marked by green triangles. The LSDV Nesterovo-2019 strains detected in Saratov Region in the current research are highlighted as the green squares.

Firstly, the phylogenetic tree based on the coding regions of the GPCR gene of the relevant nucleotide sequences of the CapPVs currently available in the NCBI database was generated using the Neighbor-Joining method (Maximum Composite Likelihood). The tree consisted of three different clusters specific for LSDV, GTPV and SPPV lineages (Figure 1), similarly to some early reports [4,31,34]. Two main clusters within the LSDV strains corresponding to wild field and the so-called ‘vaccine-like’ variants were designated as Type I and Type II, respectively (Figure 1). Each of these two general types was divided into several separate subtypes, namely four ones designated as Ia, Ib, Ic, Id, and seven subtypes as IIa, IIb, IIc, IId, IIe, IIf and IIg, respectively (Table S2 in Supplementary Material).

**Figure 2:**
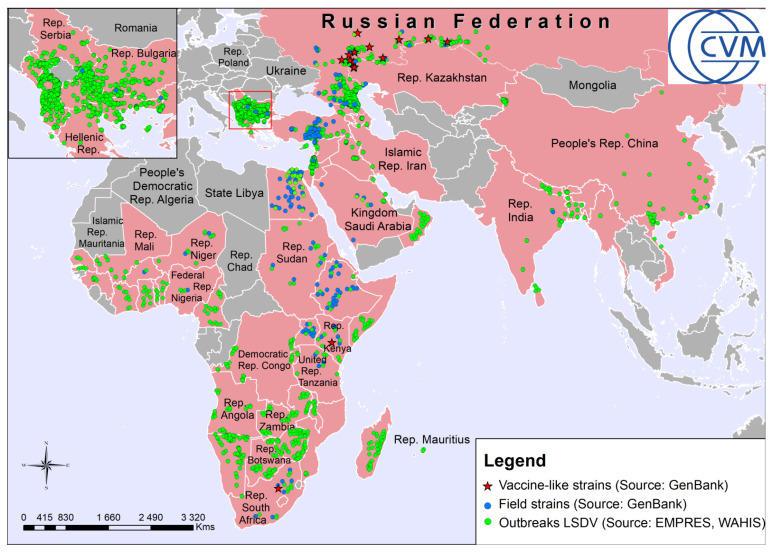
The LSD outbreaks documented worldwide in 2006–2020 (http://empres-i.fao.org/ (accessed on 2 June 2021), https://wahis.oie.int (accessed on 2 June 2021)) are highlighted as the green circles. The territories affected with LSD outbreaks are colored in pink, and those, which are officially LSD-free are in grey. The LSDV wild field and ‘vaccine-like’ strains as designated by some reports [31,36,40] are highlighted as the blue circles and red stars, respectively. The LSDV strains were discriminated into wild field and ‘vaccine-like’ variants based on the GPCR gene polymorphism as recommended [4,11,12] based on the relevant nucleotide sequences available in NCBI database (https://www.ncbi.nlm.nih.gov/ (accessed on 2 June 2021)). The maps were generated with Esri ArcGis Desktop 10.6.1 (www.esri.com (accessed on 2 June 2021)).

A molecular phylogenetic analysis of the GPCR gene sequences derived from Case1–Case3 (Saratov/Nesterovo-30614/2019/n 1, Saratov/Nesterovo-30614/2019/bl 1, Saratov/Nesterovo-30839/2019/bl 2, Saratov/Nesterovo-30839/2019/n 2, Saratov/Nesterovo-30840/2019/n 3, Saratov/Nesterovo-30840/2019/bl 3 (Nesterovo-2019 strains) proved that all the strains detected belonged to the LSDV lineage of CapPVs. Moreover, the sequences of the Case1–Case3 (afterwards Nesterovo-2019 strains) derived from specimens obtained from either blood or noses were absolutely identical to each other and belonged to a single cluster of Type II (Figure 1). This branch was formed by either the attenuated derivative strains based on the pre-1960 South African field isolate of a Neethling-type [1] such as AF409138 Neethling strain LW 1959; KX764644 Neethling-Herbivac strain; KX764645 Neethling-LSD strain-OBP and KX764643 SIS-Lumpyvax, and the South Africa field isolate of 1954 (FJ869376 RSA/54 Haden isolate LSDV17), or the more recent active outbreak-associated isolates of: (i) the 1990s from South Africa [38] (MN636839 LSD-103-GP-RSA-1991, MN636838 LSD-58-LP-RSA-1993, MN636841 LSD-220-1-NW-RSA-1993, MN636842 LSD-220-2-NW-RSA-1993, MN636840 LSD-248-NW-RSA-1993, MN636843 LSD-148-GP-RSA-1997); (ii) 2011 from Kenya (MK302071 Embu/B338/2011); (iii) 2017–2019 from four different regions of the Russian Federation, namely Samara (MH753583 Samara/2017, MK765550 Samara/1462/2018), Orenburg (MH753585 Orenburg/2017), the Republic of Bashkortostan (MH753584 Bashkortostan/2017) and the Udmurt Republic (MT134042 LSDV/Russia/Udmurtiya/2019); and (iv) 2016, Croatia, the LSDV strain that was isolated in 2016 from cattle after vaccination with a live attenuated homologous vaccine of the Neethling-Type (MG972412 Cro2016) [25,43] as well. All these strains, including Nesterovo-2019, demonstrated identical SNPs in the 22 of 27 variable positions of the GPCR gene sequence in comparison with the field strains of Type I Subtype ‘a’ or, in short, Type Ia (Table S2 in Supplementary Material). Only the LSDV strains from South Africa [38] showed a single additional SNP as substitution of A instead C in position 32 (Figure S2 in Supplementary Material) resulting a non-synonymous substitution of the relevant amino acid (Aspartic acid instead Alanine), although these strains formed a common cluster together with all strains of Type IIa (Figure 1). Type Ia LSDV strains were detected earlier (in 2015–2018) in a number of different regions of the Russian Federation, namely in: (i) 2015, the North Caucasian Regions: the Republic of North Ossetia-Alania (KY595106 RNOA-15), the Republic of Dagestan (MH893760 LSDV/Russia/Dagestan/2015), the Chechen Republic (MK765530 Chechnya/2015); (ii) 2016, the same North Caucasian Region: the Chechen Republic (MK765535 Chechnya/2016), the Republic of Ingushetia (MK765536 Ingushetiya/2016), the Kabardino-Balkarian Republic (MK765538 Kabardino-Balkariya/2016), the Karachay-Cherkess Republic (MK765537 Karachaevo-Cherkessiya/2016); the South of the European Part of Russia, the Rostov Region (MK765541 Rostov/2016); the South-East of the European Part of Russia, the Republic of Kalmykia (MK765539 Kalmykiya/2016); the West of the European Part of Russia, Tambov Region (MK765540 Tambov/2016), and Ryazan Region (MK765542 Ryazan/2016), ect.; (iii) 2017, the South-East of the European Part of Russia and the Volga Region, Saratov Region (MK432597 Saratov field/2017), and Volgograd Region, (MK432599 Volgograd/2017), and Orenburg Region (MK432598 Orenburg/2017); (iv) 2018, Samara Region (MK765548 Samara/1461/2018), and the West Siberian Plain, Kurgan Region (MK603182 LSDV/Kurgan/2018). Moreover, two LSDV strains of this Type Ia were found in 2016 during the LSD outbreak in Atyrau Region [44,47], the West Part of the Republic of Kazakhstan (MN642592 Kubash/KAZ/16 and MK765544 Kazakhstan/2016). Additionally, this Type Ia of the LSDV strains (Table S2 in Supplementary Material) was successfully identified during a number of LSD outbreaks in 1954–2020 worldwide (Figure 2).

Furthermore, there was a pronounced discrimination between the sequences of Nesterovo-2019 strains and the relevant regions of the LSDV isolates which formed three small clusters corresponding to Types Ib–d. Thus, in addition to the 22 abovementioned SNPs, the GPCR sequences of Nesterovo-2019 strains similarly to Type Ia strains had G at position 716 instead of T in both Types Ib and Ic and A at position 82 instead of T as in Type Ic (Figure S2, Table S2 in Supplementary Material). Fewer differences were found between the GPCR sequences of Nesterovo-2019 strains and the same genome regions of the LSDV recombinant strains of Types IIb-g, which were detected recently during active outbreaks of LSD in the Russian Federation only, including Saratov Region (Types IIc, d and g). Only a single SNP was detected in Nesterovo-2019 strains as a substitution of C→T in position 528 in comparison with the LSDV strain of Type IIb, and two SNPs as substitutions of G→A and C→T in positions 227 and 228, respectively, compared with the strains of Types IIc and IId (MH029290 Dergachevskyi). Additionally, the strain of Type IId showed a substitution of A instead of G in a position 360 in Nesterovo-2019 strains. At least three SNPs, such as T→C, C→T and T→C in positions 492, 528 and 555, respectively, were revealed in the GPCR sequences of Nesterovo-2019 strains compared with the strain of Type IIe (MT395337 Tymen/2019). The latter also demonstrated the substitution of C instead of A in the sequence of Nesterovo-2019 strains. Furthermore, six SNPs were detected in Nesterovo-2019 strains in comparison with the strain of Type IIf (T395338 Omsk/2019), such as T→C, A→C, A→G, G→A, G→A and C→T in positions 18, 33, 153, 159, 227 and 228, respectively. More SNPs, 11 in total, were found between the GPCR of Nesterovo-2019 strains and Type IIg strain (MK358808 Khvalynsky). SNPS detected were A instead of G, T→C, A→G, C→T, T→C, C→T, G→C, C→G, A→C, C→A and T→C in positions 227, 228, 381, 394, 400, 822, 852, 983, 987, 991 and 1050, respectively.

A comparative analysis of the GPCR gene sequences derived from Nesterovo-2019 strains demonstrated no 12-bp deletion that was considered to be typical for wild field but not for attenuated derivatives [4,32,33] of the field isolate of Neethling-type [1]. In fact, Nesterovo-2019 strains had no deletion, similar to a number of LSDV attenuated derivatives (AF409138 Neethling strain LW 1959, KX764644 Neethling-Herbivac strain, KX764645 Neethling-LSD strain-OBP), and the so-called ‘vaccine-like’ recombinant isolates (MH646674 LSDV/Russia/Saratov/2017, MH753586 Saratov/2017, MT395339 Saratov/2019, MH029290 Dergachevskyi, MT395337 Tymen/2019, MT395338 Omsk/2019 and MK358808 Khvalynsky), that were recently detected in Saratov Region and a few other regions of the Russian Federation during active LSD outbreaks in 2017–2019 (Figure S2 in Supplementary Material). In contrast, this 12-bp deletion in the position 87–98 was regularly found in the relevant GPCR sequences of the majority of outbreak-related wild field LSDV strains of Type Ia–c, except some LSDV isolates, such as the virulent Neethling variant (AF325528 Neethling-2490) and two LSDV variants from two Russian regions, Samara and Kurgan (MK765548 Samara/1461/2018 and MK603182 LSDV/Kurgan/2018). However, all LSDV isolates of Type Id (*n* = 3) also demonstrated no deletions in the GPCR gene sequence similarly to Nesterovo-2019 strains (Figure S2 in Supplementary Material).

A molecular phylogenetic analysis of the representative complete genome sequences of the LSDV strains showed that they could be also clustered into two groups corresponding to Type I, field LSDV strains and Type II, combining both vaccine and ‘vaccine-like’ or outbreak-related ones (Figure S3 in Supplementary Material). However, there was significantly less discrimination inside each of the Type resulting in only three different Subtypes, namely Ia, IIa and IIc.

### 2.4. Phylogeographic Analysis

**Figure 3:**
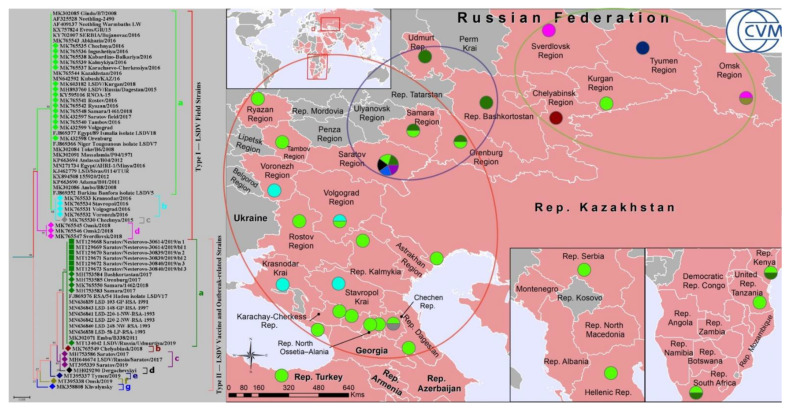
Phylogeography of the LSDV strains of Types Ia–d and IIa–g (on the **right**). The picture was constructed based on the polymorphisms of the LSDV GPCR genes obtained from the NCBI database, https://www.ncbi.nlm.nih.gov/ (accessed on 2 June 2021) (the number of cases and outbreaks and date of their registration are not specified). The phylogenetic tree (on the **left**) shows the relationships of the Nesterovo-2019 strains and other LSDV isolates as a result of typing with this scheme, with Bayesian PP values above branches, and ML bootstraps below. The map demonstrates a distribution and proportions of each Type and Subtype of LSDV in different regions of Russian Federation and worldwide. The LSDV strains of different Subtypes are highlighted as the color circles: light green for Ia, light blue—Ib, grey—Ic, pink—Id, dark green—IIa, burgundy—IIb, purple—IIc, black—IId, dark blue—IIe, olive—IIf and glowing blue—IIg. The LSDV strains are nominally combined into three clusters, depending on the territory in which they were detected, namely: (i) the Central European Part of Russia (Cluster I); (ii) the South-East of the European Part of Russia (Cluster II); (iii) the North Asian Part of Russia (Cluster III). The Cluster 1 included mainly Type I strains, while both Clusters 2 and 3 were presented by predominantly Type II strains. The Clusters 1 and 2 were partially overlapped, while the Cluster 3 did not. The Clusters are highlighted as the color ellipses: red—for Cluster I, lilac—for Cluster II, and green—for Cluster III, respectively.

The LSD outbreaks documented worldwide since 2006 are shown in Figure 2. No visible discrimination was seen between Nesterovo-2019 and other LSDV strains, which are now partially divided into main groups, such as wild field isolates, vaccine strains, and the so-called ‘vaccine-like’ recombinant variants [31,36]. In contrast, the phylogeographic analysis clearly demonstrated a relationship between Nesterovo-19 strains and the LSDV isolates, which were detected only in four Russian regions, namely Samara, Orenburg, Rep. Bashkortostan and Udmurt Rep., adjusting each other from west to east along the southern border of the Russian Federation with the Republic of Kazakhstan (Figure 3).

Overall, the Russian LSDV strains could be nominally combined into three clusters, depending on the territory in which they were detected, namely: (i) the Central European part of Russia (Cluster I); (ii) the South-East of the European part of Russia (Cluster II); (iii) the North Asian part of Russia (Cluster III). Notably, Cluster I included mainly Type I strains, while both Clusters II and III were presented by predominantly Type II strains. Clusters I and II were partially overlapped, while Cluster III did not.

More recently, the wild field isolates with a 100% homology of the GPCR sequences to Nesterovo-2019 strains were found during LSD outbreaks in South Africa in 1954 (FJ869376 RSA/54 Haden isolate LSDV17), then in 2011 in Kenya (MK302071 Embu/B338/2011), and in 2016 in Croatia as the post-vaccinal outcome [25,43]. This Haden-Type lineage was registered in Saratov Region for the first time, because the other four out of five LSD outbreaks in this territory during 2017–2019 were caused by other LSDV Subtypes (Table 2). In addition to the Haden-Type lineage, there were: (i) the Neethling wild Type Ia lineage; (ii) the recombinant Saratov/2017 IIc lineage; (iii) the specific Dergachevskyi IId lineage and (iv) the Khvalynsky IIg lineage. Thus, Nesterovo-2019 strains apparently were part of novel introduction into Saratov Region and some other Russian territories as well.

In fact, the Republic of Kazakhstan is closely located (the minimum distance to the Southeast border is 98.64 km) to the investigated Nesterovo Village of Saratov Region and some other Russian regions, such as: Volgograd, Samara, Orenburg, Kurgan, Tyumen and Omsk (Figure 2 and Figure 3). However, only a single LSDV strain was detected in Saratov Region in 2017, belonging to the same Type Ia that was identified in the Republic of Kazakhstan in 2016 [44].

## 3. Discussion

In this study we investigated the strains found in the samples from two cattle, Case1 and Case2, which were recently, about 3 weeks before sampling, vaccinated with SPPV-based vaccine. The main question was whether the cattle vaccinated against LSD were infected with LSDV as a result of incomplete protection induced by SPPV vaccine, or whether there were only certain vaccine-related side-effects associated with the inoculation with the live SPPV vaccine strain. On the one hand, the clinical manifestations of the disease were typical and indicative for LSD, such as: apathy, loss of appetite, fever, lymphadenopathy, diffuse nodular skin lesions, salivation, lachrymation and multiple nasal discharges, and pronounced respiratory problems. Moreover, both vaccinated cattle, Case1 and Case2, demonstrated identical clinical features with those observed in unvaccinated animal of Case3. However, the molecular detection and characteristic of the causative agent of the investigated cattle was critical for precise diagnostics, epidemiology of the pathogen and evaluation of some basic signs of SPPV-based vaccine, such as protective potency and possible side-effect(s). In fact, serological identification followed by differentiation with intra-specific typing is not possible due to dramatic cross-reactions between LSDV, SPPV and GTPV as the strains of CapPVs were found to be antigenically indistinguishable [21]. Since both SPPV and LSDV are phylogenetically distinct and could be differentiated using molecular tools available [1,2,3,4,35], the individual DNA from clinical specimens of Case1 and Case2 animals were carefully investigated. For this purpose, the coding region of the CapPV GPCR gene was determined according to the recommendation of Le Goff et al. [4], which has been used worldwide for successful CapPVs intra-specific differentiation.

No outbreaks of SPPV- and GTPV-related diseases in Saratov Region (https://www.fsvps.ru/fsvps-docs/ru/iac/ook/2019/12-31/ook_rf.pdf (accessed on 2 June 2021)), which could lead to any difficulty in the differentiation between SPPV vaccine and SPPV and GTPV field strains, were documented in 2019. As expected, the LSDV DNA was only detected in all the specimens of both Case1 and Case2, which implied the absence of a detectable concentration of the SPPV vaccine-related genetic materials in the cattle blood and nasal discharge. Thus, there was no SPPV co-infection in these cattle. However, the SPPV post-vaccination side effects were earlier reported after the introduction of SPPV strains with insufficient attenuation still virulent for cattle [22], which can certainly argue in favor of the assumption that it is not possible to entirely exclude the possibility of post-vaccination complications after cattle inoculation in the cases of heterologous vaccine. Moreover, no whole genome sequence of the used SPPV vaccine strain, as well as no data of clinical trials and vaccine safety for cattle as recommended (OIE, Office International des Epizooties, 2018), were available [3]. The lack of such data does not allow assessing the main parameters of the heterologous vaccine used, its safety for vaccinated animals and the vaccine strain characteristics as well. Nevertheless, the results of the current research could clearly indicate that the SPPV-based vaccine did not induce enough prompt and complete protection in cattle in the field conditions.

All six LSDV strains detected in both vaccinated Case1 and Case2 and unvaccinated Case3 cattle were identical to each other. However, the additional Case3 was dated in the same Village a month earlier. Apparently, Case3 could be the most probable source of LSD infection for Case1 and Case2 because there were no other LSD cases registered in the distance of more than 100 km. Unfortunately, it was not possible to establish the causative source for Case3. Although any known factor [1] could be involved in the LSD outbreak, none was identified under the current research. Nevertheless, the data obtained clearly proved that the SPPV-based vaccine used in Saratov Region in 2019 was unable to induce a complete protection in cattle against LSD, which is in good agreement with the previous reports [22,23,24].

Importantly, Nesterovo-2019 variants belonged to Type IIa LSDV strains (Table S2 in Supplementary Material), which was comprised of both outbreak-related and Neethling-vaccine strains (Figure 1). The latter were found in cattle after mass vaccination with the relevant live homologous LSDV vaccines providing post-vaccination complications and ‘Neethling disease’ [1]. In fact, this phenomenon could be in agreement with more recent observation on an insufficient attenuation some Neethling-based vaccine strains that might be still virulent for cattle [33].

Both vaccinated cattle of Case1 and Case2 developed a detectable serological reaction (Table 1), indicating the bovine host immune response to either the SPPV-based vaccine-associated antigens or LSD pathogen itself. Unfortunately, there was no possibility to distinguish this serological response in this study due to the high cross-reactivity between LSDV, SPV and GTPV, despite the fact that CapPV double-antigen ELISA used has been found to be an excellent tool for a rapid and simple serological examination of LSDV-vaccinated or infected cattle [2]. Nevertheless, this finding could clearly indicate the ability of cattle to elicit the early serological immune response in cattle after a single injection with SPPV-based vaccine, while the unvaccinated cow of Case3 demonstrated no detectable level of the relevant specific antibody (Table 1). In fact, the cattle of Case1 and Case2 had at least two contacts with CapPV, i.e., (i) a primary immunization with the live SPPV-based vaccine, and (ii) the infection with LSDV resulting in LSD that could be a natural boost for the bovine immune system of both animals. There were almost 3 weeks between the inoculation of SPPV-based vaccine and the development by the cattle clinical manifestations typical for LSD that could be enough to induce a detectable antibody response which was not yet protective.

In contrast, the unvaccinated cow of Case3, probably had only a single contact with the pathogen which was not enough to generate the adequate humoral immune response. On the other hand, the absence of serologic reaction could be explained by the individual reaction of the cow of Case3 to the LSDV reflecting in overall a relatively low level of sero-prevalence in calves either vaccinated against LSD or experimentally infected with LSDV [48]. However, it needs to be investigated more carefully in our future research.

Another important finding of our research was a pronounced biodiversity of the LSDV strains of both field and the so-called recombinant ‘vaccine-like’ variants, which were detected worldwide including different regions of the Russian Federation and Saratov Region too [31,36,39]. This phenomenon was discovered when the GPCR genes of Nesterovo-2019 strains and the relevant sequences of other LSDV strains available in the NCBI database were compared. Overall, the strains were divided into eleven Subtypes with four groups belonging to Type I (Subtypes a–d), and seven groups forming Type II (Subtypes a–g). However, all these LSDV strains from Type I and Type II, except several vaccine strains of IIa Subtype, were outbreak-related strains. This led us to the following conclusions: (i) the GPCR gene variability is a strong indicator of the molecular diversity of LSDV outbreak-related strains; (ii) the GPCR gene polymorphism enables-the intra-specific typing and sub-typing of LSDV outbreak-associated strains currently separated into eleven variants worldwide including those seen in the Russian Federation; (iii) the greatest GPCR polymorphism was found for the Russian strains, which demonstrated eleven different lineages (Types Ia–d and IIa–g,) in contrast to LSDV strains that were detected in other countries (Types Ia and IIa only); (iv) the GPCR is not essential for attenuation of the LSDV vaccines as it was reported previously [33], since the phylogenetic analysis did not reveal differences between the LSDV vaccine and outbreak-related strains (Figure 1 and Figure 3) that is in good correspondence with the recent report of El-Tholoth M and El-Kenawy [33] indicating that vaccines against LSD are not dependent on mutations in the GPCR gene for the attenuation; (v) the ‘novel’ recombinant strains represent the group of heterologous variants of at least six different subtypes (IIb–g) formally combined to the same phylogenetic cluster of Type II; (vi) a 12-bp deletion may not be regularly detected in wild field LSDV strains as it was reported earlier [4,32,33]; (vii) five separate lineages of LSDV strains were detected in Saratov Region during 2017–2019 since the first introduction of LSDV in 2017.

Overall, similarly to GPCR, two main Types, Type I and Type II, were formed when representative complete genome sequences of the LSDV strains were compared. Unfortunately, it was less informative than the GPCR analysis resulting in only three subtypes instead of eleven ones as were found in the GPCR (Figure S3 in Supplementary Material). This means that currently GPCR could be considered as the best appropriated target for both inter- and intra-specific discrimination of LSDV strains. These data are in agreement with some observations that GPCR gene possesses most pronounced nucleotide polymorphism than other critical genes of CapPVs [24,31,49].

Importantly, the LSDV vaccine-like strains detected in Saratov Region under the current research differed from the LSDV field strains which were reported in the bordering Republic of Kazakhstan [44,47]. In fact, Saratov Region and the Republic of Kazakhstan territories are so closely located that it could result in the possible movement of individual ruminants, including cattle, along a relatively long border. It means that the Kazakhstan LSDV field strains belonging to Type Ia may not have been the source for the current LSD outbreak in Nesterovo Village.

The data obtained proved the marked biodiversity and continued evolution of the LSDV strains detected in the Russian Federation during the last several years. In fact, since 2017 the majority of such LSDV strains with multiple SNPs have been found among the ‘vaccine-like’ strains that were outbreak-associated LSDV isolates only. It is quite possible that it could be the result of the mass vaccination of cattle with SPPV-based vaccine accompanied by the appearance of the ‘vaccine-like’ LSDV strains of Type II a–f. In fact, the ‘vaccine-like’ recombinant isolate LSDV Saratov has been reported here since 2017 [31,36]. On the contrary, no marked discrimination of LSDV strains after the annual mass vaccination of cattle with homologous vaccine(s) based on attenuated LSDV strains was reported [1]. Moreover, the LSDV-based vaccines provided the absence of any LSD outbreaks in the affected territories of Southeastern Europe [1]. However, more observation is still required to unravel the causes and mechanisms of the emergence of recombinant strains and, in general, such a pronounced biodiversity of LSDV variants that was found in this study. Overall, the scheme for typing and subtyping of LSDV strains based on the GPCR gene polymorphism used in the current study could be a useful tool for their precise molecular discrimination. The critical characteristics of the LSDV variants are: (i) derived from cattle inoculated with either homologous or heterologous vaccines against LSD and other source; (ii) found in LSD outbreak(s) in some transboundary territories; and (iii) showing accelerated molecular evolution of LSDV that has been observed worldwide in recent years.

## 4. Materials and Methods

### 4.1. Setting, Animals and Data Collection

The study was conducted on the territory of Saratov Region, Ershov District, Nesterovo Village (Latitude: 51°26′28″, Longitude: 47°57′27″), 98.4 km northwest of the nearest border between the Russian Federation and Kazakhstan (Figure S1 in Supplementary Material). Nesterovo Village has an area of 2.29 km^2^ (length—2.66 km, width—862.2 m). Two severely affected >6-month-older cattle (Case1 and Case2, respectively) were identified on 8 October 2019, following the report of a local outbreak by a small farm holder and a local qualified veterinarian (who represented the State Veterinary Regional Service). The diagnosis was based on a set of typical visible clinical manifestations of LSD, namely apathy, loss of appetite, fever, lymphadenopathy, diffuse nodular skin lesions, salivation, lachrymation and multiple nasal discharges, and pronounced respiratory problems. Additionally, a single LSD case was retrospectively revealed on a small farm in the same Village in a 6-month-older individual cow (Case3) with identical clinical features. The distance between the farms of Case1, Case2 and Case3 was about 2.4 km. All the cattle were housed indoors without mixing of herds. The owners denied any contacts between Case1 and Case2 and Case3. All of these three cases were successfully confirmed positive by real-time PCR using a commercial kit ‘Vetskrin-(Lumpy skin disease virus)’ according to the relevant protocol (Litech Company, Russia). The individual blood and nasal discharge specimens carefully obtained from each of the affected cattle (six specimens in total) were collected aseptically in sterile cryovials as described by the OIE [3]. Then, the specimens were transferred to FRCVM for further investigation. The sampled animals of Case1 and Case2 were previously (on 22 September 2019) treated against LSDV with the live dry cultural sheep pox strain vaccine (SPPV-based vaccine), Strain/serotype: ‘ARRIAH’ (Federal Center for Animal Health, Vladimir, Russia, http://www.arriah.ru/en/main/production/price-vaccines/114/cultural-dry-virus-vaccine-against-sheep-pox-and-lumpy-skin (accessed on 2 June 2021)) [1], and the cow of Case3 had no immunization against LSD with this or any other vaccine. Before vaccination and on the day when the SPPV-based vaccine was introduced, both cattle of Case1 and Case2 were healthy with no clinical signs of LSD or any other disease.

### 4.2. DNA Extraction, PCR and Sequence Evidence of LSDV

Total DNA was extracted from clinical specimens (the blood and nasal discharge samples) of the investigated cattle using the DNeasy Blood and Tissue Kit (QIAGEN GmbH, Hilden, Germany) according to the manufacturer’s instructions and then subjected to the CapPVs GPCR gene targeting PCR to identify and discriminate LSDV, SPPV and GTPV using the primers and protocol designed by Le Goff et al. [4] and Ireland and Binepal [50]. For this purpose, two primers (5′-TTAAGTAAAGCATAACTCCAACAAAAATG-3′ and 5′-TTTTTTTATTTTTTATCCAATGCTAATACT-3′) were used for the amplification of the entire GPCR gene. Amplification was done under the following conditions: initial denaturation cycle at 95 °C for 2 min, 40 cycles (denaturation at 95 °C for 45 s, annealing at 58 °C for 45 s, and extension at 72 °C for 1 min), followed by a final extension cycle at 72 °C for 10 min. The final reaction volume was prepared according to the protocol for Phusion High-Fidelity DNA Polymerase (NEB, Ipswich, MA, UK), and included 30 µL of mixture containing 1 µL (10 pmol) of forward and reverse primers each, 1 µL of sample DNA, 6 µL of 5X Phusion HF Buffer (NEB, UK), 1 µL of 10 mM dNTPs (Thermo Scientific, Waltham, MA, USA), 0.3 µL of Phusion High-Fidelity DNA Polymerase (NEB, UK), and Nuclease-Free Water (Sigma, St. Louis, MO, USA) to volume. Further, 7 µL of amplified amplicons were separated on 2% ethidium bromide-stained agarose gel electrophoresis at 100 V for 30 min and visualized in ultraviolet light transilluminator using Fast Ruler Low Range DNA Ladder (Thermo Scientific). The total DNA from a clinically healthy cow (Cow4) that had been previously immunized with SPPV-based vaccine was used as a negative control. The positive PCR products were used for a Sanger method following alignment with the 78 reference nucleotide sequences of the GPCR gene which had earlier been deposited in the NCBI database and listed in Table S1 in Supplementary Material. A phylogenetic analysis of the GPCR gene sequences derived from Case1–Case3 was performed using the Neighbor-Joining method in MEGA 7 [46]. Strain clustering and SNP analyses were performed as described [4] to define the relationships between strains at the microevolutionary level. All the representative GPCR gene sequences derived from Case1, Case2 and Case3 and reported in this research are now available in the NCBI database (Acc. No. MT129668-MT129673).

### 4.3. Detection of Specific Antibodies

To evaluate seroconversion in the Case1–Case3 animals, the commercially available ELISA kit ‘ID Screen^®^ Capripox Double Antigen Multi-Species’ (ID.vet, Montpellier, France) was used according to the manufacturer’s instructions. For this purpose, the sera from the investigated animals were routinely obtained from the relevant blood samples treated with anticoagulant following centrifuging at 1300× *g* for 10 min. Then the samples were aliquoted and stored at −20 °C until examination. The serum from a clinically healthy cow (Cow4) with a negative PCR response that had been previously immunized with SPPV-based vaccine was used as a positive control.

### 4.4. Cartographical and Phylogeographic Analysis

The surveillance data on LSD outbreaks worldwide since 2006 were collected from EMPRES Global Animal Disease Information System of the United Nations Food and Agricultural Organization (FAO, Rome, Italy) using the actual geographic coordinates for each event (https://www.oie.int/wahis_2/public/wahid.php/Diseaseinformation/Immsummary/listoutbreak (accessed on 2 June 2021)). The reported LSD outbreaks on the territory of the Russian Federation were found in the database (http://empres-i.fao.org/ (accessed on 2 June 2021)). All the representative GPCR gene sequences of the LSDV strains detected in different regions of the Russian Federation in 2015–2020, including those reported in this research and isolated in different world regions in 1954–2020 (Table S1 in Supplementary Material) were selected from the NCBI database (https://www.ncbi.nlm.nih.gov/ (accessed on 2 June 2021)). All the representative LSDV complete genome sequences were obtained from the NCBI database (https://www.ncbi.nlm.nih.gov/ (accessed on 2 June 2021)). Esri ArcGis Desktop 10.6.1 (www.esri.com (accessed on 2 June 2021)) was used for the cartographical and spatiotemporal analysis of the LSD outbreaks in certain regions of the Russian Federation and worldwide.

## 5. Conclusions

In this study, molecular typing of the LSDV strains detected in cattle immunized with SPPV vaccine during the last LSD outbreak in Saratov Region, the Nesterovo Village, in September–October, 2019, was successfully performed. For this the clinical specimens of seropositive cattle with detectable clinical manifestations of LSD, which had been previously vaccinated with the SPPV vaccine, were carefully investigated. A phylogenetic analysis based on the coding regions of the GPCR gene of the LSDV strains found in Nesterovo and the relevant nucleotide sequences of other CapPVs demonstrated their identity to the Haden-Type lineage that was historically registered first in 1954 in South Africa. The GPCR-based Typing and Subtyping was able to identify five separate lineages of LSDV strains that were detected in Saratov Region in 2017–2019, since the first introduction of LSDV in 2017. Overall, using this approach at least eleven LSDV lineages were revealed worldwide. This scheme is a promising tool for the molecular discrimination of LSDV strains derived from cattle inoculated with either homologous or heterologous vaccines against LSD, as well as for molecular epidemiology. Our further research will be aimed to carefully study the possibility of using CapPVs whole genomes in stratifying outbreaks based on their geographical location.

## Figures and Tables

**Table 1:** Detection of LSDV DNA in clinical specimens of affected cattle by the GPCR gene targeting PCR and LSDV specific antibody in ELISA.

Subject	Vaccination Status ^1^	PCR Results with Specimens from		Detection of DNA			ELISA
		Blood	Nose	LSDV	SPPV	GTPV	
Case1	vaccinated	+	+	+	−	−	+
Case2	vaccinated	+	+	+	−	−	+
Case3	unvaccinated	+	+	+	−	−	−
Cow4 (negative control)	vaccinated	−	−	−	−	−	+

^1^ SPPV-based vaccine was used for the immunization of the cattle; ‘+’—positive reaction; ‘−’—negative reaction.

**Table 2:** Five different Subtypes/lineages of LSDV strains detected in Saratov Region in 2017–2019.

Subtype	LSDV Strain ID	Date of the First Issue of the Strain	Country/Region of Identification	Date of Detection in Saratov Region	Source
Ia	AF325528 Neethling-2490	1958	Kenya	2017	[11]
IIa	FJ869376 RSA/54 Haden isolate LSDV17	1954	South Africa	2019, Ershov District	This study
IIc	MH646674 LSDV/ Russia/Saratov/2017 MH753586 Saratov/2017 MT395339 Saratov/2019	2017, 2019	The Russian Federation, Saratov Region	2017, 2019	[31,36]
IId	MH029290 Dergachevskyi	2017	The Russian Federation, Saratov Region, Dergachyovsky District	2017	NCBI Acc. No. MH029290
IIg	MK358808 Khvalynsky	2018	The Russian Federation, Saratov Region, Khvalynsky District	2018	NCBI Acc. No. MK358808

## Data Availability

Data presented in this study are available upon request.

## Supplementary Materials

The following are available online at https://www.mdpi.com/article/10.3390/pathogens10060716/s1, Figure S1: Location of the study area is in the Saratov Region which is colored in dark grey. Ershov District is highlighted in pink with Nesterovo Village labeled as a red dot. The regions of Russian Federation in close proximity with Saratov Region are in grey; the territory of the Republic of Kazakhstan is in light grey, Figure S2: Multiple sequence alignment of the LSDV GPCR gene sequences derived from the LSDV strains detected in Saratov Region in 2019 (Nesterovo-2019) and the GPCR gene sequences of other LSDV strains available at the NCBI database (https://www.ncbi.nlm.nih.gov/ (accessed on 2 June 2021)) and presented in Tables S1 and S2 in Supplementary Material. The sequences were divided into eleven Subtypes with four groups belonging to Type I (Subtypes a–d), and seven groups forming Type II (Subtypes a–g). The SNP in position 32 in the LSDV GPCR gene sequences derived from the South Africa LSDV isolates in 1990s is highlighted as the green frame, Figure S3: The phylogenetic tree of the LSDV strains of Types Ia and IIa,c. The picture was constructed based on the polymorphisms of the LSDV whole genome sequences obtained from the NCBI database, https://www.ncbi.nlm.nih.gov/ (accessed on 2 June 2021). The tree was generated using the Neighbor-Joining method in MEGA 7 [25] with branch lengths in the same units as those of the evolutionary distances used to infer the phylogenetic tree. The evolutionary distances were computed using the Maximum Composite Likelihood method [27] and are in the units of the number of base substitutions per site. The LSDV strains of different Types/Subtypes are highlighted by the color square brackets: light green for Ia (LSDV Field Strains), dark green—IIa and burgundy—IIc (LSDV vaccine and outbreak-related strains), Table S1: List of the LSDV strains available in GenBank used in this study, Table S2: Typing and Subtyping of the LSDV strains based on the coding region of the GPCR gene.

## References

[1] Calistri P., De Clercq K., Gubbins S., Klement E., Stegeman A., Abrahantes J.C., Marojevic D., Antoniou S., Broglia A. (2020). Lumpy skin disease epidemiological report IV: Data collection and analysis. EFSA J..

[2] Moller J., Moritz T., Schlottau K., Krstevski K., Hoffmann D., Beer M., Hoffmann B. (2019). Experimental lumpy skin disease virus infection of cattle: Comparison of a field strain and a vaccine strain. Arch. Virol..

[3] OIE (World Organisation for Animal Health) (2018). Lumpy skin disease. Terrestrial Manual.

[4] Le Goff C., Lamien C.E., Fakhfakh E., Chadeyras A., Aba-Adulugba E., Libeau G., Tuppurainen E., Wallace D.B., Adam T., Silber R. (2009). Capripoxvirus G-protein-coupled chemokine receptor: A host-range gene suitable for virus animal origin discrimination. J. Gen. Virol..

[5] Babiuk S., Bowden T.R., Boyle D.B., Wallace D.B., Kitching R.P. (2008). Capripoxviruses: An emerging worldwide threat to sheep, goats and cattle. Transbound. Emerg. Dis..

[6] Tuppurainen E.S., Oura C.A. (2012). Review: Lumpy skin disease: An emerging threat to Europe, the Middle East and Asia. Transbound. Emerg. Dis..

[7] Tuppurainen E.S.M., Venter E.H., Shisler J.L., Gari G., Mekonnen G.A., Juleff N., Lyons N.A., De Clercq K., Upton C., Bowden T.R. (2017). Review: Capripoxvirus Diseases: Current Status and Opportunities for Control. Transbound. Emerg. Dis..

[8] Woods J.A. (1988). Lumpy skin disease—A review. Trop. Anim. Health Prod..

[9] Weiss K.E. (1968). Lumpy skin disease virus. Virol. Monogr..

[10] Beard P.M. (2016). Lumpy skin disease: A direct threat to Europe. Vet. Rec..

[11] Tulman E.R., Afonso C.L., Lu Z., Zsak L., Kutish G.F., Rock D.L. (2001). Genome of lumpy skin disease virus. J. Virol..

[12] Tulman E.R., Afonso C.L., Lu Z., Zsak L., Sur J.H., Sandybaev N.T., Kerembekova U.Z., Zaitsev V.L., Kutish G.F., Rock D.L. (2002). The genomes of sheeppox and goatpox viruses. J. Virol..

[13] Thomas A.D. (1945). Mare CVE. Knopvelsiekte. J. S. Afr. Vet. Assoc..

[14] Diesel A.M. The epizootiology of lumpy skin disease in South Africa. Proceedings of the 14th International Veterinary Congress.

[15] Tuppurainen E.S.M., Venter E.H., Coetzer J.A.W. (2005). The detection of lumpy skin disease virus in samples of experimentally infected cattle using different diagnostic techniques. Onderstepoort J. Vet..

[16] Hunter P., Wallace D. (2001). Lumpy skin disease in southern Africa: A review of the disease and aspects of control. J. S. Afr. Vet. Assoc..

[17] MacDonald R.A.S. (1931). Pseudo-Urticaria of Cattle.

[18] Mercier A., Arsevska E., Bournez L., Bronner A., Calavas D., Cauchard J., Falala S., Caufour P., Tisseuil C., Lefrancois T. (2018). Spread rate of lumpy skin disease in the Balkans, 2015–2016. Transbound. Emerg. Dis..

[19] Miranda M.A., Stegeman J.A., Bicout D., Botner A., Butterworth A., Calistri P., Winckler C. (2016). Urgent advice on lumpy skin disease EFSA Panel on Animal Health and Welfare. EFSA J..

[20] Sevik M., Dogan M. (2017). Epidemiological and Molecular Studies on Lumpy Skin Disease Outbreaks in Turkey during 2014–2015. Transbound. Emerg. Dis..

[21] Kitching R.P. (2003). Vaccines for lumpy skin disease, sheep pox and goat pox. Dev. Biol..

[22] Tuppurainen E.S., Pearson C.R., Bachanek-Bankowska K., Knowles N.J., Amareen S., Frost L., Henstock M.R., Lamien C.E., Diallo A., Mertens P.P. (2014). Characterization of sheep pox virus vaccine for cattle against lumpy skin disease virus. Antivir. Res..

[23] Hamdi J., Bamouh Z., Jazouli M., Boumart Z., Tadlaoui K.O., Fihri O.F., El Harrak M. (2020). Experimental evaluation of the cross-protection between Sheeppox and bovine Lumpy skin vaccines. Sci. Rep..

[24] Agianniotaki E.I., Tasioudi K.E., Chaintoutis S.C., Iliadou P., Mangana-Vougiouka O., Kirtzalidou A., Alexandropoulos T., Sachpatzidis A., Plevraki E., Dovas C.I. (2017). Lumpy skin disease outbreaks in Greece during 2015–16, implementation of emergency immunization and genetic differentiation between field isolates and vaccine virus strains. Vet. Microbiol..

[25] Bedekovic T., Simic I., Kresic N., Lojkic I. (2018). Detection of lumpy skin disease virus in skin lesions, blood, nasal swabs and milk following preventive vaccination. Transbound. Emerg. Dis..

[26] Agianniotaki E.I., Chaintoutis S.C., Haegeman A., Tasioudi K.E., De Leeuw I., Katsoulos P.D., Sachpatzidis A., De Clercq K., Alexandropoulos T., Polizopoulou Z.S. (2017). Development and validation of a TaqMan probe-based real-time PCR method for the differentiation of wild type lumpy skin disease virus from vaccine virus strains. J. Virol. Methods.

[27] Menasherow S., Rubinstein-Giuni M., Kovtunenko A., Eyngor Y., Fridgut O., Rotenberg D., Khinich Y., Stram Y. (2014). Development of an assay to differentiate between virulent and vaccine strains of lumpy skin disease virus (LSDV). J. Virol. Methods.

[28] Vidanovic D., Sekler M., Petrovic T., Debeljak Z., Vaskovic N., Matovic K., Hoffmann B. (2016). Real-Time Pcr Assays for the Specific Detection of Field Balkan Strains of Lumpy Skin Disease Virus. Acta Vet. Beogr..

[29] Lamien C.E., Le Goff C., Silber R., Wallace D.B., Gulyaz V., Tuppurainen E., Madani H., Caufour P., Adam T., El Harrak M. (2011). Use of the Capripoxvirus homologue of Vaccinia virus 30 kDa RNA polymerase subunit (RPO30) gene as a novel diagnostic and genotyping target: Development of a classical PCR method to differentiate Goat poxvirus from Sheep poxvirus. Vet. Microbiol..

[30] Erster O., Rubinstein M.G., Menasherow S., Ivanova E., Venter E., Sekler M., Kolarevic M., Stram Y. (2019). Importance of the lumpy skin disease virus (LSDV) LSDV126 gene in differential diagnosis and epidemiology and its possible involvement in attenuation. Arch. Virol..

[31] Sprygin A., Pestova Y., Bjadovskaya O., Prutnikov P., Zinyakov N., Kononova S., Ruchnova O., Lozovoy D., Chvala I., Kononov A. (2020). Evidence of recombination of vaccine strains of lumpy skin disease virus with field strains, causing disease. PLoS ONE.

[32] Gelaye E., Belay A., Ayelet G., Jenberie S., Yami M., Loitsch A., Tuppurainen E., Grabherr R., Diallo A., Lamien C.E. (2015). Capripox disease in Ethiopia: Genetic differences between field isolates and vaccine strain, and implications for vaccination failure. Antiviral. Res..

[33] El-Tholoth M., El-Kenawy A.A. (2016). G-Protein-Coupled Chemokine Receptor Gene in Lumpy Skin Disease Virus Isolates from Cattle and Water Buffalo (*Bubalus bubalis*) in Egypt. Transbound. Emerg. Dis..

[34] Biswas S., Noyce R.S., Babiuk L.A., Lung O., Bulach D.M., Bowden T.R., Boyle D.B., Babiuk S., Evans D.H. (2020). Extended sequencing of vaccine and wild-type capripoxvirus isolates provides insights into genes modulating virulence and host range. Transbound. Emerg. Dis..

[35] Ochwo S., VanderWaal K., Ndekezi C., Nkamwesiga J., Munsey A., Witto S.G., Nantima N., Mayanja F., Okurut A.R.A., Atuhaire D.K. (2020). Molecular detection and phylogenetic analysis of lumpy skin disease virus from outbreaks in Uganda 2017–2018. BMC Vet. Res..

[36] Kononov A., Byadovskaya O., Kononova S., Yashin R., Zinyakov N., Mischenko V., Perevozchikova N., Sprygin A. (2019). Detection of vaccine-like strains of lumpy skin disease virus in outbreaks in Russia in 2017. Arch. Virol..

[37] Sprygin A., Van Schalkwyk A., Shumilova I., Nesterov A., Kononova S., Prutnikov P., Byadovskaya O., Kononov A. (2020). Full-length genome characterization of a novel recombinant vaccine-like lumpy skin disease virus strain detected during the climatic winter in Russia, 2019. Arch. Virol..

[38] Van Schalkwyk A., Kara P., Ebersohn K., Mather A., Annandale C.H., Venter E.H., Wallace D.B. (2020). Potential link of single nucleotide polymorphisms to virulence of vaccine-associated field strains of lumpy skin disease virus in South Africa. Transbound. Emerg. Dis..

[39] Aleksandr K., Pavel P., Olga B., Svetlana K., Vladimir R., Yana P., Alexander S. (2020). Emergence of a new lumpy skin disease virus variant in Kurgan Oblast, Russia, in 2018. Arch. Virol..

[40] Sprygin A., Pestova Y., Prutnikov P., Kononov A. (2018). Detection of vaccine-like lumpy skin disease virus in cattle and *Musca domestica* L. flies in an outbreak of lumpy skin disease in Russia in 2017. Transbound. Emerg. Dis..

[41] Salnikov N., Usadov T., Kolcov A., Zhivoderov S., Morgunov Y., Gerasimov V., Gogin A., Titov I., Yurkov S., Malogolovkin A. (2018). Identification and characterization of lumpy skin disease virus isolated from cattle in the Republic of North Ossetia-Alania in 2015. Transbound. Emerg. Dis..

[42] OIE (World Organisation for Animal Health) World Animal Health Information Database (WAHIS) Interface. https://www.oie.int/wahis_2/public/wahid.php/Diseaseinformation/Immsummary.

[43] Lojkic I., Simic I., Kresic N., Bedekovic T. (2018). Complete Genome Sequence of a Lumpy Skin Disease Virus Strain Isolated from the Skin of a Vaccinated Animal. Genome Announc..

[44] Mathijs E., Vandenbussche F., Saduakassova M., Kabduldanov T., Haegeman A., Aerts L., Kyzaibayev T., Sultanov A., Van Borm S., De Clercq K. (2020). Complete Coding Sequence of a Lumpy Skin Disease Virus Strain Isolated during the 2016 Outbreak in Kazakhstan. Microbiol. Resour. Announc..

[45] OIE (Office International des Epizooties) World Organisation for Animal Health. https://www.oie.int.

[46] Kumar S., Stecher G., Tamura K. (2016). MEGA7: Molecular Evolutionary Genetics Analysis Version 7.0 for Bigger Datasets. Mol. Biol. Evol..

[47] Issimov A., Rametov N., Zhugunissov K., Kutumbetov L., Zhanabayev A., Kazhgaliyev N., White P. (2020). Emergence of the First Lumpy Skin Disease Outbreak Among Livestock in the Republic of Kazakhstan in 2016. Preprints.

[48] Haegeman A., De Leeuw I., Mostin L., Van Campe W., Aerts L., Vastag M., De Clercq K. (2020). An Immunoperoxidase Monolayer Assay (IPMA) for the detection of lumpy skin disease antibodies. J. Virol. Methods.

[49] Byadovskaya O., Pestova Y., Kononov A., Shumilova I., Kononova S., Nesterov A., Babiuk S., Sprygin A. (2020). Performance of the currently available DIVA real-time PCR assays in classical and recombinant lumpy skin disease viruses. Transbound. Emerg. Dis..

[50] Lkjlkj Ireland D.C., Binepal Y.S. (1998). Improved detection of capripoxvirus in biopsy samples by PCR. J. Virol. Methods.

