# Supplementary material for: Genetic Evidence of Multiple Introductions of Lumpy Skin Disease Virus into Saratov Region, Russia
Source: Pathogens. 2021 Jun 7;10(6):716. doi: 10.3390/pathogens10060716 (PMC8227815; doi:10.3390/pathogens10060716)
Supplement: Supplementary file 1 [file pathogens-10-00716-s001.zip › pathogens-1234127-supplementary.pdf]

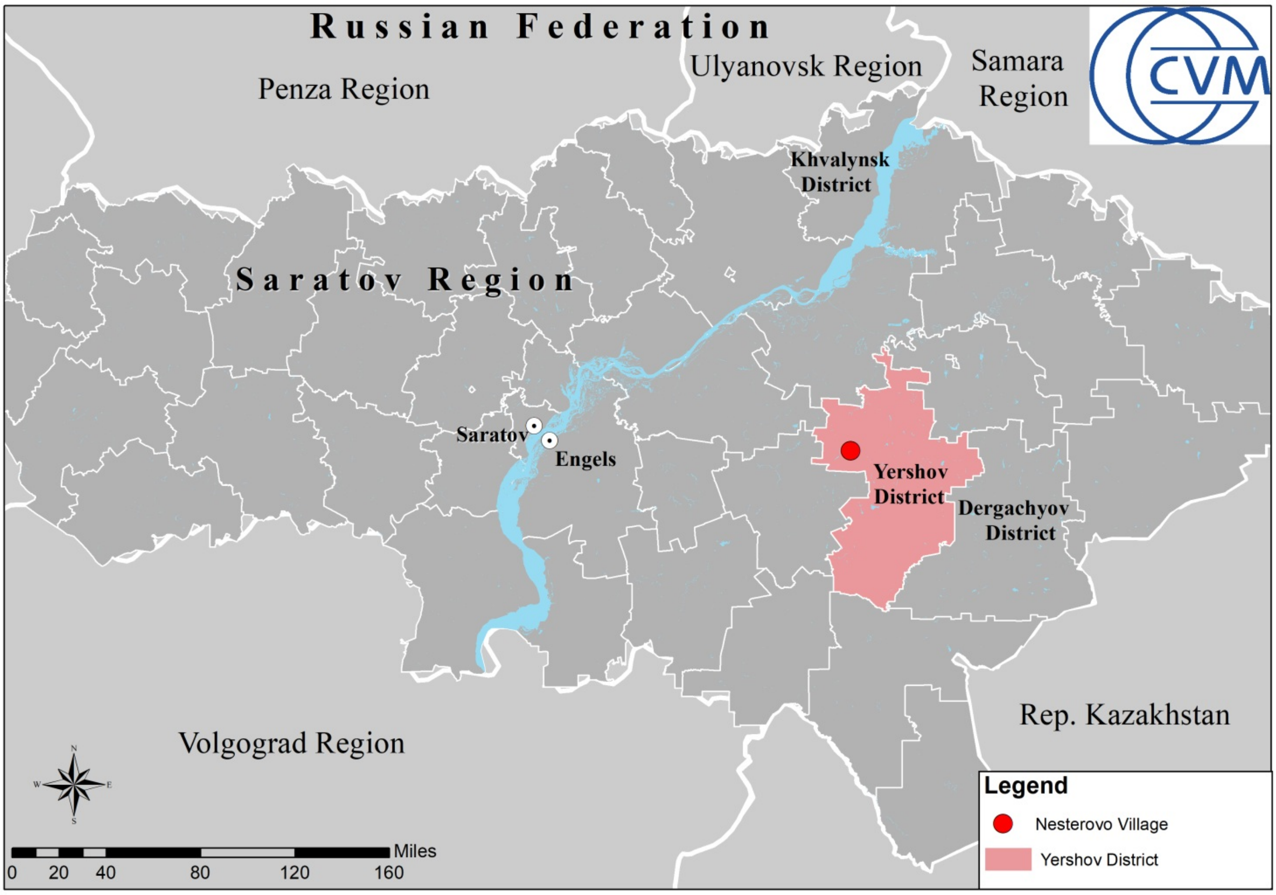

Figure S1. Location of the study area is in the Saratov Region which is colored in dark grey. Ershov District is highlighted in pink with Nesterovo Village labeled as a red dot. The Regions of Russian Federation in close proximity with Saratov Region are in grey; the territory of the Republic of Kazakhstan is in light grey.

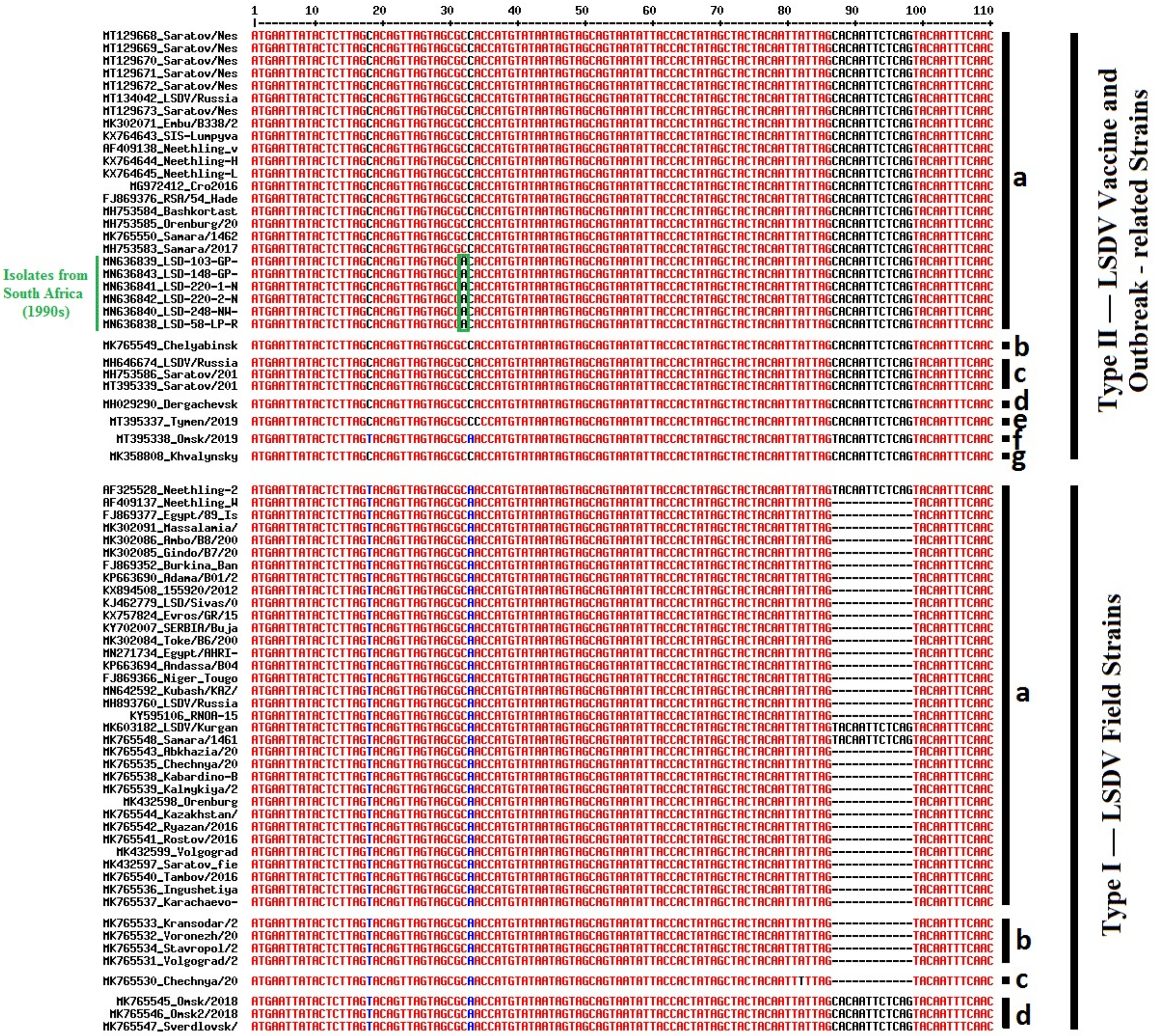

Figure S2. Multiple sequence alignment of the LSDV GPCR gene sequences derived from the LSDV strains detected in Saratov Region in 2019 (Nesterovo-2019) and the GPCR gene sequences of other LSDV strains available at the NCBI database (https://www.ncbi.nlm.nih.gov/) and presented in Table S1 and Table S2 in Supplementary Material. The sequences were divided into eleven Subtypes with four groups belonging to Type I (Subtypes a-d), and seven groups forming Type II (Subtypes a-g). The SNP in position 32 in the LSDV GPCR gene sequences derived from the South Africa LSDV isolates in 1990s is highlighted as the green frame.

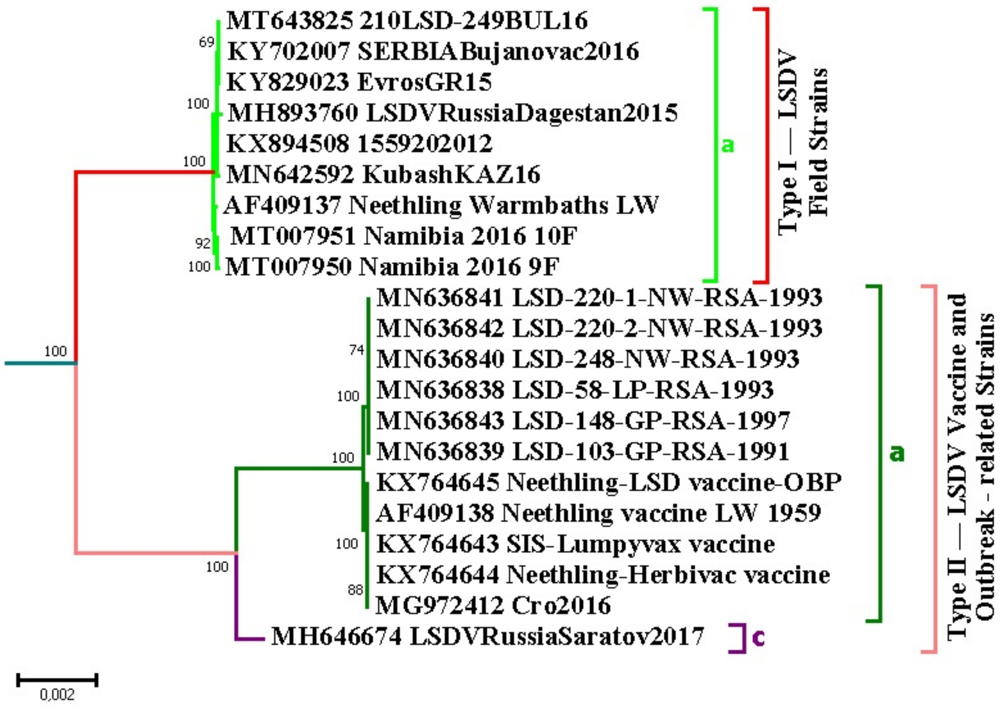

Figure S3. The phylogenetic tree of the LSDV strains of Types Ia and IIa,c. The picture was constructed based on the polymorphisms of the LSDV whole genome sequences obtained from the NCBI database, https://www.ncbi.nlm.nih.gov/. The tree was generated using the Neighbor-Joining method in MEGA 7 (25) with branch lengths in the same units as those of the evolutionary distances used to infer the phylogenetic tree. The evolutionary distances were computed using the Maximum Composite Likelihood method (27) and are in the units of the number of base substitutions per site. The LSDV strains of different Types/Subtypes are highlighted by the color square brackets: light green for Ia (LSDV Field Strains), dark green – IIa and burgundy – IIc (LSDV vaccine and outbreak-related strains).

Table S1. List of the LSDV strains available in GenBank used in this study.

##	STRAIN NAME	GENBANK ACCESSION	COLLECTION DATE	HOST	COUNTRY
1	Bashkortastan/2017	MH753584	09/2017	Cattle	Russia
2	Chechnya/2015	MK765530	-N/A-	Unknown	-N/A-
3	Chechnya/2016	MK765535	-N/A-	Unknown	-N/A-
4	Chelyabinsk/2018	MK765549	-N/A-	Unknown	-N/A-
5	Dergachevskyi	MH029290	06/2017	Cattle	Russia
6	Kabardino-Balkariya/2016	MK765538	-N/A-	Unknown	-N/A-
7	Kalmykiya/2016	MK765539	-N/A-	Unknown	-N/A-
8	Karachaevo-Cherkessiya/2016	MK765537	-N/A-	Unknown	-N/A-
9	Khvalynsky	MK358808	09/2018	Cattle	Russia
10	Kransodar/2016	MK765533	-N/A-	Unknown	-N/A-
11	Ingushetiya/2016	MK765536	-N/A-	Unknown	-N/A-
12	LSDV/Kurgan/2018	MK603182	-N/A-	Unknown	-N/A-
13	LSDV/Russia/Dagestan/2015	MH893760	2015	Bos taurus	Russia
14	LSDV/Russia/Saratov/2017	MH646674	2017	Bos taurus	Russia
15	Omsk/2018	MK765545	-N/A-	Unknown	-N/A-
16	Omsk2/2018	MK765546	-N/A-	Unknown	-N/A-
17	Orenburg	MK432598	2017	Cow	Russia
18	Orenburg/2017	MH753585	12/2017	Cattle	Russia
19	RNOA-15	KY595106	10/2015	Cattle	Russia North Ossetia-Alania
20	Rostov/2016	MK765541	-N/A-	Unknown	-N/A-
21	Ryazan/2016	MK765542	-N/A-	Unknown	-N/A-
22	Samara/1461/2018	MK765548	-N/A-	Unknown	-N/A-
23	Samara/1462/2018	MK765550	-N/A-	Unknown	-N/A-
24	Samara/2017	MH753583	07/2017	Cattle	Russia
25	Saratov/2017	MH753586	07/2017	Cattle	Russia
26	Saratov_field/2017	MK432597	2017	Cattle	Russia
27	Stavropol/2016	MK765534	-N/A-	Unknown	-N/A-
28	Sverdlovsk/2018	MK765547	-N/A-	Unknown	-N/A-
29	Tambov/2016	MK765540	-N/A-	Unknown	-N/A-
30	Volgograd	MK432599	2017	Cow	Russia

31	Volgograd/2016	MK765531	-N/A-	Unknown	-N/A-
32	Voronezh/2016	MK765532	-N/A-	Unknown	-N/A-
33	LSDV/Russia/Udmurtiya/2019	MT134042	03/03/2019	Cattle	Russia
34	Omsk/2019	MT395338	2019	Cattle	Russia
35	Saratov/2019	MT395339	2019	Cattle	Russia
36	Saratov/Nesterovo-30614/2019/bl_1	MT129669	2019	Cattle	Russia
37	Saratov/Nesterovo-30614/2019/n_1	MT129668	2019	Cattle	Russia
38	Saratov/Nesterovo-30839/2019/bl_2	MT129671	2019	Cattle	Russia
39	Saratov/Nesterovo-30839/2019/n_2	MT129670	2019	Cattle	Russia
40	Saratov/Nesterovo-30840/2019/bl_3	MT129673	2019	Cattle	Russia
41	Saratov/Nesterovo-30840/2019/n_3	MT129672	2019	Cattle	Russia
42	Tymen/2019	MT395337	2019	Cattle	Russia
43	Kazakhstan/2016	MK765544	-N/A-	Unknown	-N/A-
44	Kubash/KAZ/16	MN642592	07/07/2016	Bos taurus	Kazakhstan
45	Abkhazia/2016	MK765543	-N/A-	Unknown	-N/A-
46	Adama/B01/2011	KP663690	10/02/2011	Cattle	Ethiopia
47	Andassa/B04/2012	KP663694	10/11/2012	Cattle	Ethiopia
48	Massalamia/P04/1971	MK302091	1971	Cattle	Sudan
49	Evros/GR/15	KX757824	08/18/2015	Cattle	Greece
50	Embu/B338/2011	MK302071	2011	Cattle	Kenya
51	Neethling 2490	AF325528	-N/A-	Unknown	Kenya
52	Egypt/AHRI-1/Minya/2016	MN271734	2016	Cattle	Egypt
53	Egypt/89 Ismalia isolate LSDV18	FJ869377	1989	Cattle	Egypt
54	155920/2012	KX894508	12/19/2012	Cattle	Israel
55	LSD/Sivas/0114/TUR	KJ462779	01/06/2014	Cattle	Turkey
56	Gindo/B7/2008	MK302085	2008	Cattle	Tanzania
57	Ambo/B8/2008	MK302086	2008	Cattle	Tanzania
58	Toke/B6/2008	MK302084	2008	Cattle	Tanzania

59	SERBIA/Bujanovac/2016	KY702007	06/07/2016	Cattle	Serbia
60	Neethling Warmbaths LW	AF409137	-N/A-	Cattle	South Africa
61	RSA/54 Haden isolate LSDV17	FJ869376	1954	Cattle	South Africa
62	Niger Tougounous isolate LSDV7	FJ869366	-N/A-	Cattle	Niger
63	Burkina Banfora isolate LSDV5	FJ869352	-N/A-	Cattle	Burkina Faso
64	Neethling vaccine LW 1959	AF409138	-N/A-	-N/A-	South Africa
65	Neethling-Herbivac vaccine	KX764644	-N/A-	Bos taurus	South Africa
66	Neethling-LSD vaccine-OBP	KX764645	-N/A-	Bos taurus	South Africa
67	SIS-Lumpyvax vaccine	KX764643	1999	Bos taurus	South Africa
68	LSD-103-GP-RSA-1991	MN636839	1991	Bos taurus	South Africa
69	LSD-148-GP-RSA-1997	MN636843	1997	Bos taurus	South Africa
70	LSD-220-1-NW-RSA-1993	MN636841	1993	Bos taurus	South Africa
71	LSD-220-2-NW-RSA-1993	MN636842	1993	Bos taurus	South Africa
72	LSD-248-NW-RSA-1993	MN636840	1993	Bos taurus	South Africa
73	LSD-58-LP-RSA-1993	MN636838	1993	Bos taurus	South Africa
74	Cro2016	MG972412	2016	Cattle	Croatia
75	Abu Gharib (Sheeppox virus)	MN072626	-N/A-	Sheep	Iraq
76	Jaipur (Sheeppox virus)	MG000156	1981	Sheep	India
77	FZ (Goatpox virus)	KC951854	01/10/2012	Goat	China
78	India (Goatpox virus)	MN072620	-N/A-	Goat	-N/A-

Table S2. Typing and Subtyping of the LSDV strains based on the coding region of the GPCR gene.

Type	Subtype	Strain name (Acc. number of identical sequences in GenBank)[1]	SNP in the position of the LSDV GPCR nucleotide sequence																										
			**18**	**32**	**33**	**34**	**82**	**153**	**159**	**227**	**228**	**360**	**381**	**394**	**400**	**492**	**528**	**555**	**648**	**716**	**803**	**822**	**849**	**852**	**983**	**987**	**991**	**1050**	**1116**
**I Field strains**	**a**	MK302086 Ambo/B8/2008<br>KP663690 Adama/B01/2011<br>MK302085 Gindo/B7/2008<br>KX894508 155920/2012<br>KJ462779 LSD/Sivas/0114/TUR<br>FJ869366 Niger Tougounous/LSDV7<br>MK302084 Toke/B6/2008<br>MK302091 Massalamia/P04/1971<br>KP663694 Andassa/B04/2012<br>MN271734 Egypt/AHRI-1/Minya/2016<br>MK432598 Orenburg<br>FJ869352 Burkina Banfora/LSDV5<br>FJ869377 Egypt/89 Ismalia/LSDV18<br>MK432599 Volgograd<br>MK432597 Saratov field/2017<br>MK765540 Tambov/2016<br>MK765542 Ryazan/2016<br>MK765541 Rostov/2016<br>MK765548 Samara/1461/2018<br>KY595106 RNOA-15<br>MH893760 LSDV/Russia/Dagestan/2015<br>MK603182 LSDV/Kurgan/2018<br>MN642592 Kubash/KAZ/16<br>MK765544 Kazakhstan/2016<br>MK765537 Karachaevo-Cherkessiya2016<br>MK765539 Kalmykiya/2016<br>MK765538 Kabardino-Balkariya/2016<br>MK765536 Ingushetiya/2016<br>MK765535 Chechnya/2016<br>MK765543 Abkhazia/2016<br>KY702007 SERBIA/Bujanovac/2016<br>KX757824 Evros/GR/15<br>AF409137 Neethling Warmbaths LW<br>AF325528 Neethling-2490	T	C	A	A	A	A	G	G	C	G	G	T	C	T	C	T	C	G	C	T	A	A	T	G	A	C	C
	**b**	MK765533 Kransodar/2016<br>MK765534 Stavropol/2016<br>MK765531 Volgograd/2016<br>MK765532 Voronezh/2016	T	C	A	A	A	A	G	G	C	G	G	T	C	T	C	T	C	T	N[1]	N	N	N	N	N	N	N	N
	**c**	MK765530 Chechnya/2015	T	C	A	A	T	A	G	G	C	G	G	T	C	T	C	T	C	T	N	N	N	N	N	N	N	N	N

		MK765546 Omsk2/2018																										
	**d**	MK765545 Omsk/2018	T	C	A	A	A	G	A	A	T	G	A	T	C	T	C	T	C	G	N	N	N	N	N	N	N	N	N
		MK765547 Sverdlovsk/2018																										
		MT129668 Saratov/Nesterovo-30614/2019/n 1																										
		MT129669 Saratov/Nesterovo-30614/2019/bl 1																										
		MT129670 Saratov/Nesterovo-30839/2019/n 2																										
		MT129671 Saratov/Nesterovo-30839/2019/bl 2																										
		MT129672 Saratov/Nesterovo-30840/2019/n 3																										
		MT129673 Saratov/Nesterovo-30840/2019/bl 3																										
		AF409138 Neethling vaccine LW 1959	C	C	C	A	A	G	A	A	T	G	A	C	T	C	T	C	T	G	T	C	C	G	C	A	C	T	T
		KX764644 Neethling-Herbivac vaccine																										
	**a**	KX764645 Neethling-LSD vaccine-OBP																										
		KX764643 SIS-Lumpyvax vaccine																										
		MG972412 Cro2016																										
		FJ869376 RSA/54 Haden isolate LSDV17																										
		MK302071 Embu/B338/2011																										
**II 'Vaccine-like' strains**		MH753583 Samara/2017																										
		MK765550 Samara/1462/2018																										
		MH753585 Orenburg/2017																										
		MH753584 Bashkortastan/2017																										
		MT134042 LSDV/Russia/Udmurtiya/2019																										
		MN636839 LSD-103-GP-RSA-1991																										
		MN636843 LSD-148-GP-RSA-1997																										
		MN636841 LSD-220-1-NW-RSA-1993	C	A	C	A	A	G	A	A	T	G	A	C	T	C	T	C	T	G	T	C	C	G	C	A	C	T	T
		MN636842 LSD-220-2-NW-RSA-1993																										
		MN636840 LSD-248-NW-RSA-1993																										
		MN636838 LSD-58-LP-RSA-1993																										
	**b**	MK765549 Chelyabinsk/2018	C	C	C	A	A	G	A	A	T	G	A	C	T	C	C	C	T	G	N	N	N	N	N	N	N	N	N
		MH646674 LSDV/Russia/Saratov/2017	C	C	C	A	A	G	A	G	C	G	A	C	T	C	T	C	T	G	T	C	C	G	C	A	C	T	T
	**c**	MH753586 Saratov/2017	C	C	C	A	A	G	A	G	C	G	A	C	T	C	T	C	T	G	N	N	N	N	N	N	N	N	N
		MT395339 Saratov/2019	C	C	C	A	A	G	A	G	C	G	A	C	T	C	T	C	T	G	N	N	N	N	N	N	N	N	N
	**d**	MH029290 Dergachevskyi	C	C	C	A	A	G	A	G	C	A	A	C	T	C	T	C	T	G	T	T	C	C	G	C	A	C	T
	**e**	MT395337 Tymen/2019	C	C	C	C	A	G	A	A	T	G	A	C	T	T	C	T	T	G	N	N	N	N	N	N	N	N	N
	**f**	MT395338 Omsk/2019	T	C	A	A	A	A	G	G	C	G	A	C	T	C	T	C	T	G	N	N	N	N	N	N	N	N	N
	**g**	MK358808 Khvalynsky	C	C	C	A	A	G	A	G	C	G	G	T	C	C	T	C	T	G	T	T	C	C	G	C	A	C	T

[1] N – Not determined, this part of the sequence is not available in the GenBank
